# Aβ-40 Y10F Increases βfibrils Formation but Attenuates the Neurotoxicity of Amyloid-β Peptide

**DOI:** 10.3390/ijms13055324

**Published:** 2012-04-25

**Authors:** Xueling Dai, Ping Chang, Wenjuan Liu, Ke Xu, Yaxuan Sun, Shigong Zhu, Zhaofeng Jiang

**Affiliations:** 1 Department of Physiology and Pathophysiology, Peking University School of Basic Medical Sciences, Beijing 100191, China; E-Mail: xueling@ygi.edu.cn; 2 Beijing Key Laboratory of Bioactive Substances and Functional Foods, Beijing Union University, Beijing 100191, China; E-Mails: changping@ygi.edu.cn (P.C.); liuali99@163.com (W.L.); sunxx@ygi.edu.cn (Y.S.); 3 College of Life Science, Capital Normal University, Beijing 100048, China; E-Mail: xuke1987@163.com

**Keywords:** Alzheimer’s disease, amyloid-β peptide, βfibrils, neurotoxicity

## Abstract

Alzheimer’s disease (AD) is characterized by the abnormal aggregation of amyloid-β peptide (Aβ) in extracellular deposits known as senile plaques. The tyrosine residue (Tyr-10) is believed to be important in Aβ-induced neurotoxicity due to the formation of tyrosyl radicals. To reduce the likelihood of cross-linking, here we designed an Aβ-40 analogue (Aβ-40 Y10F) in which the tyrosine residue was substituted by a structurally similar residue, phenylalanine. The aggregation rate was determined by the Thioflavin T (ThT) assay, in which Aβ-40 Y10F populated an ensemble of folded conformations much quicker and stronger than the wild type Aβ. Biophysical tests subsequently confirmed the results of the ThT assay, suggesting the measured increase of β-aggregation may arise predominantly from enhancement of hydrophobicity upon substitution and thus the propensity of intrinsic β-sheet formation. Nevertheless, Aβ-40 Y10F exhibited remarkably decreased neurotoxicity compared to Aβ-40 which could be partly due to the reduced generation of hydrogen peroxide. These findings may lead to further understanding of the structural perturbation of Aβ to its fibrillation.

## 1. Introduction

Alzheimer’s disease (AD), the principal cause of dementia, is characterized by neuronal degeneration, synaptic loss, the presence of senile plaques (SPs), and neurofibrillary tangles (NFTs). Extracellular SPs are composed of an amyloid-β-(Aβ)-containing core surrounded by activated microglia, fibrillary astrocytes, and dystrophic neuritis [1]. Aβ is derived via proteolytic cleavage of a ubiquitous transmembrane protein, termed the amyloid precursor protein (APP), by the action of β- and γ-secretases [2,3]. The β-amyloid cascade theory stipulates a central role of Aβ production, oligomerization and/or fibrillogenesis in triggering a cascade of cellular and molecular events leading to the process of synaptic failure, synapse loss, neuronal loss and eventually the occurrence of AD [1,4].

Aβ aggregates, including oligomers, protofibrils and fibrils, are believed to be the main causes that lead to AD [5]. Although no clear correlation has been drawn between the density of deposits and the severity of AD, previous findings by our group and other investigators suggest a negative correlation between the level of Aβ fibrils and cognitive impairment [6–9]. More and more evidence indicates that oligomers or protofibrils rather than Aβ plaques, contribute to early dendritic and synaptic injury and thereby neuronal dysfunction [10,11].

The generation of reactive oxygen species (ROS) has been linked with Aβ neurotoxicity and neurodegeneration in AD [4,12,13]. Among the proposed mechanisms involving Aβ-induced oxidative stress, tyrosine is proposed to be a critical residue for its susceptibility to oxidation, especially under condition of oxidative stress [12,13]. An Aβ peptide with tyrosine-10 replaced by alanine is reported to be non-toxic and unable to induce oxidative stress responses in cells [14]. The tyrosine residue of Aβ could form a stable tyrosyl radical during metal catalyzed oxidation (MCO), since the proximity of tyrosine to the neighboring histidine residues allows redox active metal ions to receive an electron to form a tyrosine radical [15].

Due to the comparative stability of tyrosyl radicals, tyrosine has been considered to be important in Aβ-induced neurotoxicity, the formation of Aβ aggregation and amyloid plaques [16]. Here we synthesized an Aβ-40 analogue termed Aβ-40 Y10F (tyrosine 10 replaced by phenylalanine) and explored its aggregate and neurotoxic properties. Our results indicate that the residue substitution led to an increased percent of peptides undertaking a β-sheet rich conformation but a dramatic decrease in neurotoxicity. These data may shed light upon the role of the tyrosine residue in Aβ folding and neurotoxicity, and also provide further evidence for the structural perturbation of Aβ to its fibrillation.

## 2. Results and Discussion

### 2.1. Secondary Structural Changes of Aβ (1–40) Y10F

AD is characterized by the formation of amyloid deposits composed primarily of Aβ [17,18], a natively unfolded protein with little or no ordered structure under physiological conditions [19,20]. Thus, the conditions that decrease the net charge or increase the hydrophobicity could be expected to result in partial folding. Previous studies suggest that the formation of Aβ dimers through the phenolic coupling of tyrosine (termed dityrosine-linkage) could be an early event critical to fibril formation and Aβ deposits in AD [15,16]. Murakami and colleagues reported that Aβ-42 Y10F shows significantly attenuated neurotoxicity compared to that of wild type Aβ-42 [21]. Other reports suggest that the substitution of tyrosine with alanine in the Aβ sequence inhibits the ability of this peptide to catalyze the production of H_2_O_2_ and to cross-link, as well as inhibiting Aβ neurotoxicity [14]. For these reasons, tyrosine 10 in the chain of Aβ is believed to be critical in the process of AD pathogenesis.

**Figure 1:**
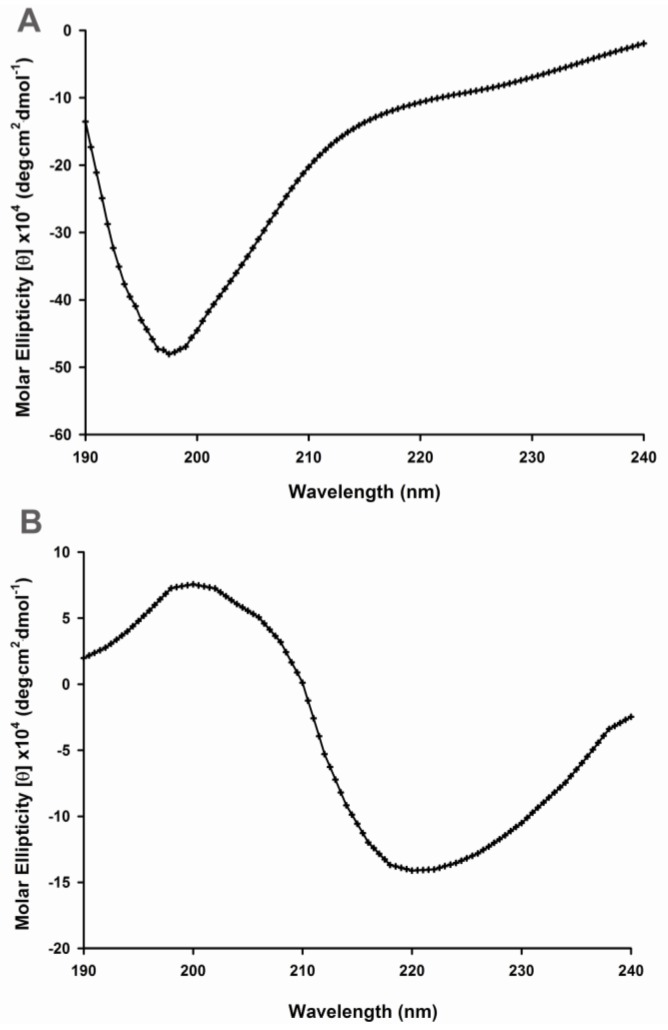
UV region circular dichroism (CD) spectra of various Aβ peptides. Molar ellipticity of wild type Aβ-40 (**A**) and Aβ-40 Y10F (**B**) was recorded in the UV region (190–240 nm). Aβ peptides were diluted using 10 mM phosphate buffer (pH 7.4) to a final concentration of 50 μM in each sample. The average data of six run is shown.

In order to investigate the relationship between amino acid residues of Aβ-40 and conformational changes, our approach was firstly to record circular dichroism (CD) spectra in the far UV region (190–240 nm) to detect the secondary structures of wild type and mutant peptides. The CD spectrum of wild type Aβ-40 was characterized by a strong negative CD band in the 195- to 200-nm region (Figure 1A), indicative of a disordered or random-coil secondary structure [8]. In contrast, the spectral curve of Aβ-40 Y10F displayed a β-sheet spectrum as characterized by a minimum at 220 nm and a positive ellipticity at ~200 nm (Figure 1B).

### 2.2. Aggregation of Wild Type Aβ (1–40) and Aβ-40 Y10F

**Figure 2:**
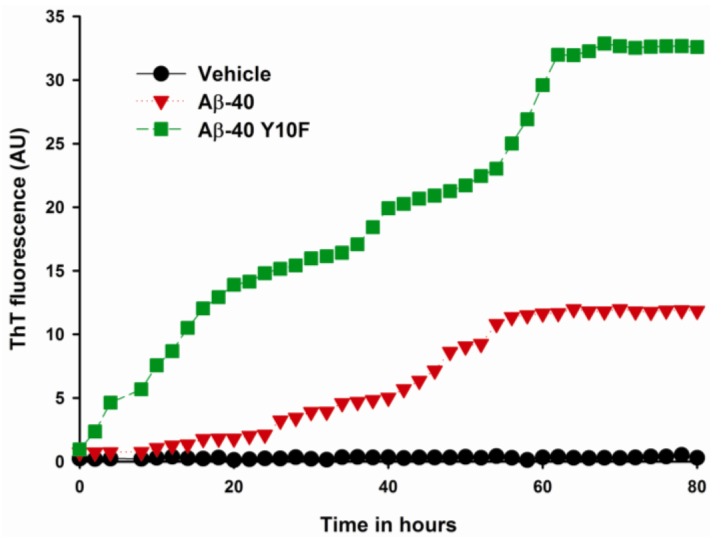
Kinetic measurements of β-aggregation resulting from incubation of wild type Aβ-40 and Aβ-40 Y10F. ThT assay was performed with 10 μM Aβ, 10 μM ThT and 10 mM phosphate buffer at 37 °C. ThT fluorescence was monitored at an excitation of 450 nm and emission of 485 nm. Each point represents the average of six independent experiments.

Aggregation is a characteristic property of partially folded proteins and most aggregating protein systems probably involve a transient partly folded intermediate as the key precursor of fibrillation [22]. To confirm that the Aβ-40 Y10F-induced CD spectrum alteration was directly related to changes in the protein conformation, we performed a kinetic aggregation study to explore the effect of Y10 on the aggregative ability through beta-sheet formation of Aβ-40. The β-aggregation of either wild type Aβ-40 or Aβ-40 Y10F was assessed using the fluorescent dye ThT. Both type of peptides exhibited a gain in fibril, as evident by an increase in ThT fluorescence during the time course. As shown in Figure 2, it was interesting to note that the aggregation of Aβ-40 Y10F showed a more pronounced rise than that of wild type Aβ-40, which could be due to the secondary structural changes of modified Aβ, leading to a greater binding capacity for ThT or an increase in the total amount of aggregation. The aggregation of wild type Aβ-40 reached a steady state at around 60 h, while Aβ-40 Y10F remained in a rapid growth phase until 72 h, suggesting a higher aggregation ability of Aβ-40 Y10F compared to that of wild-type Aβ-40.

**Figure 3:**
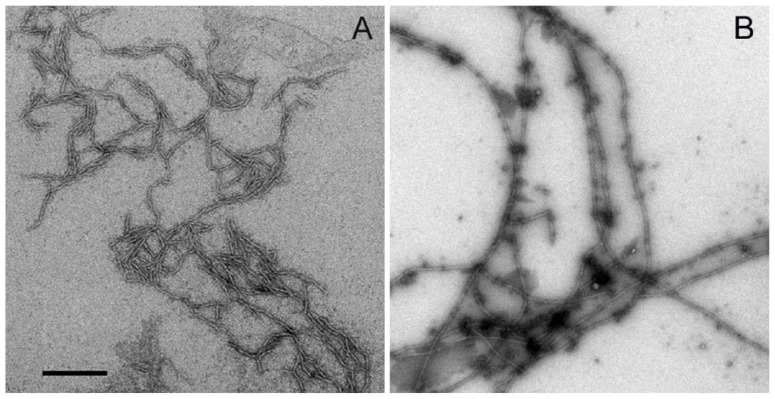
Negatively stained transmission electron micrographs of Aβ-40 (**A**) and Aβ-40 Y10F (**B**). The peptide was diluted in phosphate buffer to 10 μM, and then incubated at 37 °C for 72 h before experiments. Both peptides presented fibrillar morphologies, wild type Aβ-40 showed the combined morphologies of fibrils (5–10 nm in diameter and 400 nm in length) and curvilinear protofibrils (6–8 nm in diameter and <100 nm in length), while Aβ-40 Y10F gave rise to longer mature fibrils (long and extended fibrils >2 μm in length). Scale bar: 200 nm.

Further, peptides were separately incubated at 37 °C without agitation and allowed to fibrillize for 72 h to investigate the aggregation state of Aβ-40 and Aβ-40 Y10F. Both peptides presented fibrillar morphologies though aggregates formed in wild type Aβ-40 apparently consists of shorter fibrils and associate laterally with each, while Aβ-40 Y10F gave rise to longer mature fibrils (long, rigid, and extended fibrils that could be longer than 2 μm in length) and small amounts of spherical structures (Figure 3B). TEM analysis of the fractions of wild type Aβ-40 showed the combined morphologies of fibrils (5–10 nm in diameter and >400 nm in length) and curvilinear aggregates (6–8 nm in diameter and <200 nm in length) (Figure 3A), and the latter are consistent with previously described morphologies for protofibrils that present as dispersed, curvilinear structures of 4–11 nm diameter and <200 nm length [22–24].

The key distinguishing features of protofibrillar aggregates include that they bind to amyloid-specific dyes, such as Congo red and Thioflavin-T (ThT) though much weaker than mature fibrils, and protofibrils are less stable than mature amyloid fibrils [23,25,26]. It is anticipated that the protofibrils or shorter fibrils formed in wild type Aβ-40 are a partially folded form and could be a precursor of fibrils, since the aggregates have contiguous hydrophobic patches on the surface and are prone to foster self-association and hence potentially fibrillation [27], though the structure of wild type Aβ-40 resembles that of other reports under the same concentration of Aβ [25,26]. However, protofilaments as structural subunits of a mature fibril are distinctive from protofibrils [28]. Furthermore, the highly ordered structures such as protofibrils and Aβ-derived diffusible ligands (ADDLs) can be “on pathway” to fibril formation (disappear upon fibril formation) or “off pathway” species (they do not convert into fibrils) [29]. It is important to mention that the protofibrils of wild type Aβ-40 are unlikely to change into fibrils since the images were taken when the process of fibrillization was complete as indicated by the maximum ThT fluorescence (Figure 2). Since synthetic peptides have been shown to have slight biophysical variations in batch-to-batch preparations [30], we repeated EM experiments using three different batches of both peptides to verify the above results. Again, we observed similar conformational patterns using various batches, suggesting that the conformation of Aβ-40 and Aβ-40 Y10F were not due to an unusual synthesis of peptides.

**Figure 4:**
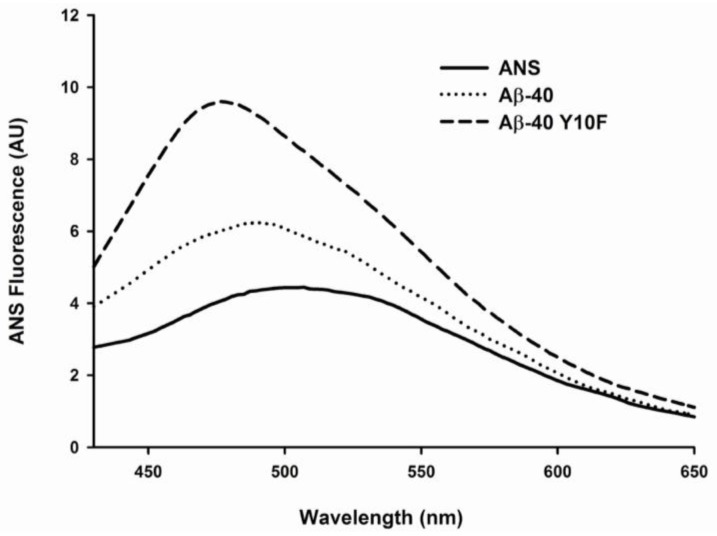
Effect of variant Aβ peptides on ANS fluorescence. Fluorescence spectra were collected from 430 to 650 nm, with excitation at 350 nm. Solid lines symbolize unbound ANS in phosphate buffer, while ANS binding to Aβ-40 and Aβ-40 Y10F are represented by dotted and dashed lines, respectively. Each point represents the average of three independent experiments.

The binding of 1-Anilinonaphthaleine-8-Sulphonic Acid (ANS) to hydrophobic surfaces of protein is represented as enhanced fluorescence and a blue shift in the wavelength of peak emission (*λ*max), as illustrated by the binding of ANS to wild type Aβ-40 or Aβ-40 Y10F in Figure 4. The results revealed enhanced ANS fluorescence in the presence of both Aβ peptides. After binding to wild type Aβ-40, there was an approximately 1.5-fold increase in quantum yield and a blue shift in maximum emission wavelength for ANS from 506 to 490 nm. By contrast, the change in the fluorescence of ANS upon binding to Aβ-40 Y10F was much more pronounced, with an approximately 2.5-fold increase in quantum yield and a blue shift in maximum emission wavelength to around 476 nm compared to unbound ANS, suggesting structural reorganization of Aβ in the form of more ordered folding when containing the tyrosine to phenylalanine substitution.

### 2.3. Tyrosine Substitution Rendered Aβ-40 Less Toxic via Reduced Generation of Hydrogen Peroxide

The aggregation and toxicity of Aβ highly correlates with its sequence and structure [31]. Previous studies demonstrated that protofibrils or oligomers, rather than Aβ fibrils, mainly contributed to early dendritic and synaptic injury and thereby neuronal dysfunction [11,32]. Using a small molecule methylene blue, Necula *et al.* successfully inhibited Aβ oligomerization concomitant with the promotion of fibrillization [33], suggesting that oligomers and fibril formation are distinct and competing pathways. According to the above biophysical experiments, Aβ-40 Y10F displayed structural changes and its capability of forming β-sheet rich conformations was apparently increased. To investigate the correlation between the peptides’ aggregation and their physiological toxicity, either Aβ-40 or Aβ-40 Y10F treated cortical neurons were tested to see if residue substitution exerted effects on neurotoxicity.

**Figure 5:**
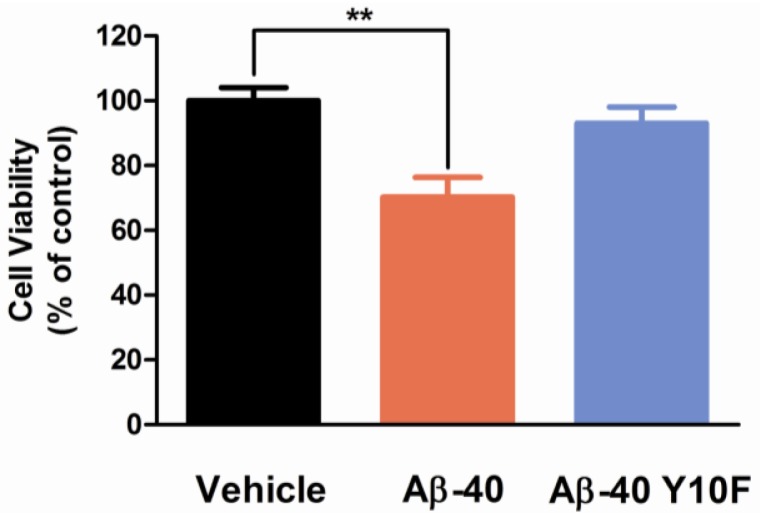
Neurotoxicity of variant Aβ peptides. Primary neuronal cells were grown for 7 days *in vitro*, and then neurons were exposed to various Aβ peptides at the final concentration of 5 μM for 96 h. Cell viability was determined by measuring the reduction of MTS. Data are presented as the mean ± S.E.M. of three independent experiments. Student’s *t* test was applied to determine the significance, denoted as ^**^ *P* < 0.01.

As determined by MTS assay, exposure to 5 μM wild type Aβ-40 for 96 h showed significantly stronger neurotoxicity than Aβ-40 Y10F, as the cell viability rate was reduced to ~70% (*P* < 0.01 *versus* Vehicle, Figure 5). It is possible that the formation of an Aβ tyrosyl radical leads to increased oligomerization via the formation of dityrosine-linkage as an early aggregation step, which is supported by the identification of dityrosine in amyloid plaque [12]. Whilst for neurons treated with 5 μM Aβ-40 Y10F for up to 96 h, the cell viability rate was ~93% (no significant difference *versus* Vehicle, Figure 5), suggesting a markedly decreased toxicity of Aβ-40 with a tyrosine substitution. This lack of neurotoxicity is in line with Murakami’s reports, which suggest the neurotoxicity of Aβ-42 Y10F was significantly attenuated compared to that of wild type Aβ-42 [21]. However, the reduced toxicity of Aβ-40 with a tyrosine→phenylalanine substitution is contrary to previous study which suggests rodent Aβ-42 (R5G, Y10F, H13R) exhibits similar neurotoxicity to human Aβ-42 [34]. The reason for the discrepancy could probably be attributed to several factors such as differences with the type of peptide employed (various amino acid sequences) or differences in peptide aggregate forms, concentrations, *etc.*

**Figure 6:**
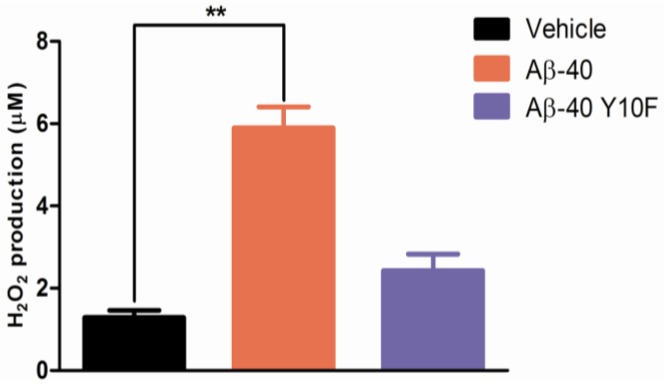
H_2_O_2_ production induced by wild type Aβ-40 or Aβ-40 Y10F. Primary cortical neurons were exposed to 5 μM wild type Aβ-40 or Aβ-40 Y10F for 96 h. Absorbance readings were taken and compared to vehicle (Student’s *t* test, ^**^ *P* < 0.01, *n* = 3).

Aβ has been shown to induce protein oxidation and lipid peroxidation [35,36], while the phenol hydroxyl group of tyrosine (a crucial residue involved in oxidative stress) is easily oxidized through metal ions to give a tyrosyl radical [12]. However, the mechanism of its formation at the molecular level has not fully been clarified. To investigate the contribution of tyrosine to the generation of free radicals, the amount of H_2_O_2_ produced by wild type Aβ-40 or Aβ-40 Y10F was examined. As shown in Figure 6, Aβ-40 Y10F produced fairly low levels of H_2_O_2_ as compared to wild type Aβ-40. These results, together with those of the cell viability assay suggest that the Aβ-40 Y10F induced low level of H_2_O_2_ generation could partly account for its low cytotoxicity provided that H_2_O_2_ is not the sole factor responsible for Aβ-induced toxicity, confirming that tyrosyl radicals play a critical role in the radical-mediated neurotoxicity of Aβ-40 [15].

## 3. Experimental Section

### 3.1. Materials

All cell culture reagents were obtained from Invitrogen (Carlsbad, USA). 1-anilinonaphthaleine-8-sulphonic acid (ANS) was obtained from Cayman Chemical Company (Michigan, USA). 1,1,1,3,3,3-hexafluoro-2-propanol (HFIP), poly-l-lysine, thioflavin T (ThT), and dimethyl sulfoxide (DMSO) were purchased from Sigma-Aldrich (St. Louis, MO, USA). CellTiter 96^®^ Aqueous One Solution Reagent was procured from Promega (Madison, WI, USA). All other chemical reagents were commercial products of analytical grade and the highest purity available.

### 3.2. Peptide Preparation

Wild type Aβ-40 and Aβ-40 Y10F were synthesized by employing solid phase Fmoc chemistry and produced by Biosynthesis Biotech Co., Ltd, Beijing, China. After removal from the resin and de-protection, samples were purified and characterized by reverse phase HPLC using UV/Vis detection, and the purity of all peptides used was at least 95%. The lyophilized peptides were initially dissolved in HFIP to a final concentration of 1 mg/mL, shaken for 2 h at 4 °C to entirely dissolve the peptide, then aliquoted into siliconized tubes and stored at −80 °C [20]. Prior to experiment, the stock solution was spin-vacuumed using the Integrated Speed-Vac System (Thermo Savant, USA) then diluted in 10 mM phosphate buffer (pH 7.4) to the required concentration.

### 3.3. Circular Dichroism Spectroscopy

Secondary structural changes in peptides were detected using CD spectroscopy [8,20]. The stock peptide was spin-vacuumed then dissolved in 10 mM phosphate buffer (pH 7.4), and the final concentration of Aβ in each sample was 50 μM. All measurements were performed in quartz cuvette cells with a pathlength of 1 mm and scanned with a J-810 CD spectropolarimeter (Jasco, Japan). CD measurements were carried out between 190 and 240 nm using the following parameters: 2-nm bandwidth, 20 nm/min run speed, 0.5-nm step size, and 2-s response time. Background values for each test were subtracted from the corresponding CDs of each sample, and the spectra were smoothed using the Jasco software FFT filter function and converted to molar ellipticity.

### 3.4. Fluorescence Spectroscopy (ThT Assay)

The relative degree of β-aggregation is determined by ThT that specifically binds to fibrillar structures [37,38]. Aβ stock solution was diluted in phosphate buffer to 10 μM, ThT was then added to each sample to a final concentration of 10 μM. Each sample was measured in terms of fluorescence intensity at 37 °C using a Varioskan multimode microplate spectrophotometer (Thermo, USA) under kinetic fluorometric mode. Measurements were performed every 2 h at an excitation wavelength of 450 nm and an emission of 485 nm [8,20]. To account for the background fluorescence, the fluorescence intensity measured from each control solution without Aβ peptide was subtracted from that of each solution containing Aβ.

### 3.5. Transmission Electron Microscopy

The solution of 10 μM Aβ peptide diluted in phosphate buffer was incubated at 37 °C for 72 h. Each sample was spotted onto glow discharged carbon-coated 300 mesh copper grids (Ted Pella Inc., Redding, CA, USA) for 1 min, dried, and then negatively stained with 2% uranyl acetate [39]. The excess was wiped off, and the grid was allowed to dry. Specimens were then examined with a JEM-1400 electron microscope (JEOL, Japan) at 80 kV accelerating voltage.

### 3.6. 1-Anilinonaphthaleine-8-Sulphonic Acid (ANS) Binding

ANS is a fluorescent dye that binds with high affinity to hydrophobic surfaces of proteins. The emission maximum of ANS undergoes a blue shift and increases significantly upon binding to the hydrophobic portions [40,41]. We measured ANS fluorescence to monitor structural changes in Aβ-40 and Aβ-40 Y10F upon 4 d aging. The final concentration of peptide in the solution was 10 μM, and the [ANS]/[Aβ] ratio in the following experiments was kept equals to 5. Emission spectra were recorded from 430 to 650 nm with excitation at 350 nm.

### 3.7. Primary Neuronal Cultures

Cortical neuronal cultures were performed as described previously with some modifications [9]. Briefly, embryonic day 17 Sprague Dawley rat cortices were dissociated and suspended in fresh neurobasal medium plus 2% B27 supplements, 1% penicillin-streptomycin, and 0.5 mM l-glutamine, then plated onto poly-l-lysine-coated 96-well culture plates at a density of 5 × 10^4^ cells per well. This method resulted in cultures highly enriched for neurons (>95% purity) with minimal astrocyte and microglial contents. The neuronal cells were allowed to mature for 7 days *in vitro* before commencing treatment. For treatment, samples were diluted to a concentration of 5 μM in serum-free neurobasal medium, aged for 72 h, and finally added to the cultures for 96 h.

### 3.8. Cell Viability Assay

Cell viability was quantitively determined using the MTS assay as described previously [42]. Briefly, cells were cultured in flat bottomed 96-well plates and exposed to various treatments. 20 μL CellTiter 96^®^ Aqueous One Solution Reagent (MTS) was added to each well according to the manufacturer’s instructions. After 4 h in culture the cell viability was determined by measuring the absorbance at 490 nm using a μQuant MQX200 reader (Bio-Tek). Background readings of MTS incubated in cell-free medium were subtracted from each value before calculations. The data were normalized and calculated as a percentage of untreated control values prior to analysis.

### 3.9. Hydrogen Peroxide Assay

Analyses were carried out using a hydrogen peroxide assay kit (Beyotime Biotech, China) as described previously [9]. Hydrogen peroxide could oxidize Fe^2+^ to Fe^3+^, and then Fe^3+^ reacted with xylenol orange resulting in a colorimetric reaction that could be further detected by a spectrometer. Briefly, test tubes containing 50 μL of supernatants and 100 μL of test solutions were placed at room temperature for 20 min and measured immediately with a spectrometer at a wavelength of 560 nm. The concentration of H_2_O_2_ released was calculated from a standard concentration curve in triplicate experiments.

## 4. Conclusions

In the present study, we employed an Aβ-40 analogue in which tyrosine was replaced by a phenylalanine residue. Despite its great structural similarity to tyrosine, phenylalanine is classified as nonpolar because of the hydrophobic nature of its benzyl side chain. In accordance with previous reports [8,9,43], the folded conformation is characterized by an increased amount of ordered secondary structure and the appearance of hydrophobic clusters. Our results suggest that wild type Aβ-40 displays a far-UV CD spectrum typical of an unfolded polypeptide chain, implying the lack of ordered secondary conformation under these conditions, while the CD spectrum of Aβ-40 Y10F was manifested as a dramatic increase in [θ]_220_ (negative CD intensity) suggesting a β-sheet structure of the peptide.

Data from the ThT assay indicated that both wild type Aβ-40 and Aβ-40 Y10F exhibit a fluorescence gain in a time-dependent manner [23], whereas Aβ-40 Y10F accelerated the kinetics of βfibril formation and resulted in a more marked rise than wild type Aβ. These results lead us to assume that the structure of wild type Aβ-40 consists mainly of intermediates to β-fibril (probably profibrils or shorter fibrils) under the current conditions, while mature fibrils are generated in solution containing Aβ-40 Y10F. Although this argument is admittedly speculative, the basic idea arising from this logic was subsequently confirmed by using TEM, in which peptides aged for 72 h were used to examine the aggregation state. Indubitably, Aβ-40 Y10F gives rise to longer fibrils than those formed in sample consisting of wild type Aβ-40. Changes in ANS fluorescence also confirmed the highly ordered hydrophobic surfaces of Aβ-40 Y10F compared to that of wild type Aβ-40. Fibrillar Aβ was initially reported to facilitate the neuronal cell death process in primary rat hippocampal cultures, and non-amyloidogenic or amorphous aggregates of Aβ are comparatively nontoxic compared with fibrillar Aβ [22,31]. Interestingly, multiple Aβ aggregated species have been identified, and neurotoxicity appears to be correlated with the amount of nonfibrillar oligomers and protofibrils [6,11,44]. Our results confirmed the latter—that Aβ-40 Y10F led to increased βfibrils conformation rendering the peptide less toxic, and this lack of neurotoxicity could be partly due to the reduced generation of hydrogen peroxide.

Former reports suggest that oligomeric fibrillization intermediates (protofibrils or oligomers), rather than the fibrils themselves, are pathogenic, though the mechanism by which they cause neuronal death remains unknown [45,46]. If Aβ oligomers or protofibrils represent the primary pathogenic species, then selective inhibition of these highly toxic species by blocking initial nucleation of Aβ rather than dissolution of amyloid fibrils or inhibiting later stages of fibrillogenesis may be an attractive means of therapeutic intervention. Studies are still on the way to elucidate the mechanism by which Aβ structures are associated with neurotoxicity, though it seems that amyloid fibrils with various structures and toxicity as well as soluble oligomers are all involved in the pathogenesis of AD in a complicated way. In conclusion, these data are expected to be of increasing importance in the future to direct or refine further model building.
